# A Next Generation Formulation of Curcumin Ameliorates Experimentally Induced Osteoarthritis in Rats *via* Regulation of Inflammatory Mediators

**DOI:** 10.3389/fimmu.2021.609629

**Published:** 2021-03-12

**Authors:** Mehmet Yabas, Cemal Orhan, Besir Er, Mehmet Tuzcu, Ali Said Durmus, Ibrahim Hanifi Ozercan, Nurhan Sahin, Prakash Bhanuse, Abhijeet Ashok Morde, Muralidhara Padigaru, Kazim Sahin

**Affiliations:** ^1^ Department of Genetics and Bioengineering, Trakya University, Edirne, Turkey; ^2^ Department of Animal Nutrition, Faculty of Veterinary Medicine, Firat University, Elazig, Turkey; ^3^ Department of Biology, Faculty of Science, Firat University, Elazig, Turkey; ^4^ Department of Surgery, Faculty of Veterinary Medicine, Firat University, Elazig, Turkey; ^5^ Department of Pathology, Faculty of Medicine, Firat University, Elazig, Turkey; ^6^ Research&Development, OmniActive Health Technologies, Mumbai, India

**Keywords:** anti-inflammatory, curcumin, immunomodulation, inflammation, inflammatory diseases, osteoarthritis, osteoimmunology

## Abstract

Osteoarthritis (OA) is a chronic and debilitating disease of the knee joint. OA of the knee is initiated by physical damage and accumulated oxidative stress, followed by an exaggerated inflammation leading to cartilage damage. Currently, no effective and safe therapeutic option capable of restoring articular cartilage tissue and joint architecture is available. We here report a novel and highly bioavailable formulation of curcumin, labeled as Next Generation Ultrasol Curcumin (NGUC), which was 64.7 times more bioavailable than natural 95% curcumin extract as demonstrated in rat bioavailability studies. We further investigated the protective effect of NGUC against monosodium iodoacetate (MIA)‐induced knee OA in rats. Analysis of X-ray and histopathological images revealed that NGUC supplementation restored joint architecture and reduced swelling of joints induced by MIA. NGUC treatment caused a significant reduction in the levels of inflammatory mediators such as TNF-α, IL-1β, IL-6, COMP, and CRP, and expressions of MMP-3, 5-LOX, COX-2, and NFκB in synovial tissue of rats with MIA-induced OA. NGUC also decreased serum MDA level and increased the levels of antioxidant enzymes SOD, CAT, and GPX. Thus, our results indicate that a novel formulation of curcumin with enhanced bioavailability effectively ameliorates the pathophysiology of OA.

## Introduction

Osteoarthritis (OA) is a degenerative joint disease and the leading cause of disability in the world. The risk of being diagnosed with OA increases with age, obesity, female sex, genetic factors and sports injuries (1–3). OA is a chronic joint disorder characterized by inflammation and articular cartilage degradation resulting in movement-associated pain. Although the involvement of wear and tear has long been considered as the primary reason for the development of OA (4), recent studies have suggested the involvement of inflammatory signaling pathways that play a role in the pathogenesis of OA (5–11). Increased levels of conventional inflammatory mediators such as tumor necrosis factor-alpha (TNF-α), interleukin (IL)-1β and IL-6, nuclear factor kappa B (NFκB), and cyclooxygenase 2 (COX-2) in synovial tissue contribute to the development of OA and have become targets for the therapeutic strategies against OA (12). Current patient management of OA is primarily dependent on dietary supplements from chondroitin sulfate, non-steroidal anti-inflammatory drugs (NSAIDs) and analgesics to provide symptomatic relief from pain (13, 14). However, the long-term use of NSAIDs is associated with adverse events such as upper gastrointestinal toxicity (15). Plant-based multi-functional anti-inflammatory products that are safer over long-term use are increasingly being considered in addition to pharmaceutical products.

Curcumin, a polyphenol derived from the rhizomes of turmeric (*Curcuma longa*), is commonly used as a spice in foods and is one of the best-studied natural agents with a broad range of biological activities including anti-inflammatory, antioxidant, and antiproliferative activities (16–18). Curcumin modulates multiple molecular targets and signaling pathways, cell cycle proteins, cytokines, and chemokines (16–18). Curcumin has been widely used for centuries with no adverse effects and hence the demand for curcumin as a source of natural product for various health benefits including OA has increased. However, clinical benefits of curcumin are limited due to its poor solubility, low absorption, and rapid metabolism in the gut (19). Only a fraction of the ingested curcumin in natural format is absorbed while the rest is excreted through the feces (20). Curcumin is also highly sensitive and hence subjected to degradation in the alkaline pH of the small intestine (21). Numerous formulation approaches have been developed to improve the bioavailability of curcumin, such as the use of adjuvant like piperine that interferes with glucuronidation, use of liposomes, nanoparticles, and formation of phospholipid complex (22).

Here we report a new curcumin formulation called Next Generation Ultrasol Curcumin (NGUC) with improved oral bioavailability and can withstand the alkaline pH of the small intestine. Further, we tested the anti-osteoarthritic effects of NGUC in a rat model after oral administration and revealed that NGUC supplementation alleviates the severity of OA by modulating inflammatory mediators and oxidative stress markers.

## Materials and Methods

### Preparation of NGUC

Standardized 95% curcuminoids extract (Akay Flavours, India), phospholipids (Vav Lipids Pvt. Ltd, India), monoglycerides (BASF, India), and acidifier (Archer Daniels Midland Company, India) were dissolved in isopropyl alcohol with stirring at 55°C –60°C for 40 min. The above solution was cooled down to 40°C, and hypromellose (Shin-Etsu Chemical Company, Japan), medium-chain triglyceride oil (AAK Kamani, India) and Tocopherol (Matrix fine sciences, India) were added, stirred for 45 min to obtain a homogeneous solution. The spray drying was carried out with a Laboratory spray dryer (LU 227 Labultima Process Technologies Pvt. Ltd, India) to obtain powder which was further dried in a vacuum dryer to obtain a yellowish-orange free-flowing homogeneous powder.

### Pharmacokinetic Study

This study was conducted at Anthem Biosciences, Bangalore, India. Adult male Sprague Dawley rats were used for this study under the supervision of Committee for the Purpose of Control and Supervision of Experiments on Animals (CPCSEA) guidelines for laboratory animals facility published in the gazette of India, December 15^th^ 1998 and in accordance with the protocol approved by Institutional Animal Ethics Committee (IAEC Protocol No: ABD/IAEC/PR/89/17-20). Animals were housed under standard laboratory conditions in an environmentally monitored air-conditioned room with adequate fresh air supply (10–15 air changes per hour), room temperature 22 ± 3°C, and relative humidity 30%–70%, with 12 h light and 12 h dark cycle. Nutrilab rodent feed (Manufactured by Provimi Animal Nutrition Pvt Ltd) and water from Aqua guard water filter cum purifier were provided *ad libitum*. The rats were fasted overnight with free access to water prior to the experiment and were divided randomly into two groups of 8 animals each. The rats were administered NGUC or 95% turmeric extract as 100% (w/v) aqueous suspension to deliver 200 mg/kg body weight equivalent of total curcuminoids. Under mild isoflurane anesthesia, the 10 blood samples were collected (0.00, 0.25, 0.50, 1.00, 2.00, 3.00, 4.00, 6.00, 8.00, and 24 h) from retro-orbital plexus up to 24 h post-dose into pre-labeled tubes containing anticoagulant (K2EDTA - 2mg/ml blood). Collected blood samples were centrifuged at 1,520 rcf, 4°C for 10 min, and plasma was separated and stored at −80°C until analysis. Fifty microliters of plasma samples were added to the tubes and vortexed. Then, 200 µl of acetonitrile containing internal standard (100 ng/ml ISD) was added, vortexed, and centrifuged at 9,500 rcf for 10 min at 10°C. The supernatant was separated and then transferred into the autosampler vials and submitted for LC-MS/MS analysis.

Quantification of three Curcuminoids analytes Curcumin, Demothoxycurcumin, and Bisdemethoxycurcumin were performed in rat plasma by LC-MS-MS analysis (API 3200 Q Trap). Chromatograms were acquired using Analyst^®^ software version 1.6.1. The concentration of the unknown sample was calculated from the following equation using regression analysis with a peak area ratio of standard and ISD vs. calibration standards concentration, using weighting factor (1/x^2^): y = mx + c, where, x = concentration of drug, m = slope of the calibration curve, y = peak area ratio, c = intercept of the calibration curve, 1/x^2^ was used as the weighting factor. Plasma pharmacokinetic parameters were determined by non-compartmental analysis using Phoenix WinNonlin 8.1 software (Certara, NJ, USA). Maximum concentration (Cmax) and time to reach maximum concentration (Tmax) values obtained directly from the concentration-time curve. The area under the concentration-time curve (AUC0-t) was also determined.

### Animals Used in MIA-Induced OA Study

Female Wistar rats (8 weeks of age, mean weight of 180 ± 200g) were purchased from Firat University Experimental Research Centre. Animals were kept in a room with controlled temperature (23 ± 2°C), humidity (55 ± 10%), light (12/12h light-dark cycle), and food/water *ad libitum* throughout the experiment. The study was approved by the Animal Ethics Committee of Firat University (2019/88-135) and done in accordance with the standard ethical guidelines for laboratory animal use and care as defined in the European Economic Community rules (EEC, 1986).

### Experimental Design

Female Wistar rats were randomly allocated into four groups (n=7 each): Control; OA group; OA+Cur 1, 100 mg/kg of NGUC (20 mg/kg of curcuminoids); OA+Cur 2, 200 mg/kg of NGUC (40 mg/kg of curcuminoids) provided by OmniActive Health Technologies, Mumbai, India as explained earlier. The rat model of knee OA was generated as described previously (23, 24). Briefly, the right knee of rats was shaved and disinfected with 70% alcohol following anaesthetization using xylazine (10 mg/kg) and ketamine hydrochloride (50 mg/kg). Three mg of MIA (Sigma, St. Louis, USA) was dissolved in 50 μl saline and injected into the right knee joints through the infrapatellar ligament using a 0.3 ml insulin syringe fitted with a 29G needle. The control group received an injection of 50 μl saline only. Two weeks after injection of MIA, NGUC suspended in 1 ml saline was orally given for four weeks at the doses of 100 mg/kg of NGUC (20 mg/kg of curcuminoids) and 200 mg/kg of NGUC (40 mg/kg of curcuminoids). Animals were observed every other day to assess knee joint swelling. Four weeks after NGUC administration, rats were sacrificed, and blood and the specimens of the knee joint were collected for the follow‐up experiments. The blood samples were centrifuged at 850 rcf for 10 min, and serum was separated from the blood and stored at −20°C for further analysis.

### Analysis of Biochemical Parameters in Serum

The levels of serum glucose, creatinine, blood urea nitrogen (BUN), total protein (TP), albumin (ALB), globulin (GLOB), alanine aminotransferase (ALT), aspartate aminotransferase (AST), alkaline phosphatase (ALP), and total bilirubin (TBIL) were analyzed using a portable automated chemistry analyzer (Samsung LABGEO PT10V, Samsung Electronics Co., Suwon, Korea). Serum TNF-α, IL-1β, IL-6, IL-10, cartilage oligomeric matrix protein (COMP), and C-reactive protein (CRP) levels were analyzed using commercially available enzyme-linked immunosorbent assay (ELISA) kits according to the manufacturer instructions (Cayman Chemical, Ann Arbor, MI, USA).

The levels of superoxide dismutase (SOD), catalase (CAT), and glutathione peroxidase (GPX) were determined using the commercially available kits according to the manufacturer instructions (Cayman Chemical, Ann Arbor, MI, USA). For malondialdehyde (MDA) analysis, an HPLC apparatus of Shimadzu UV–vis SPD-10 AVP detector, a CTO-10 AS VP column, and 30mM KH_2_PO_4_ and methanol (82.5: 17.5, v/v, pH 3.6) at a flow rate of 1.2 ml/min were used (Shimadzu, Japan). Column waste was monitored at 250 nm.

### Histological Assessment of the Knee Joint

The knee joint tissues were embedded (in paraffin) and cut into 5 μm sections. The sections were stained with hematoxylin and eosin (H&E) as previously described (25). The stained sections were examined under a microscope (Olympus, Japan). Toluidine blue (Sigma) staining was performed to evaluate proteoglycans and glycosaminoglycans in the cartilage matrix (26). Briefly, the slides were deparaffinized, hydrated and stained by 0.10%. For toluidine blue staining, the dehydrated slides were placed directly into the 0.40% toluidine blue solution (diluted in 0.1M sodium acetate buffer) for 5 min.

### Measurement of Joint Swelling (Edema)

Three measures of knee joint thickness were taken under anesthesia using an electronic digital caliper (Mitutoyo Absolute Digimatic 150 mm, Japan). The results were expressed as an average in mm.

### Gait Test

The hind paws of rats were brushed with ink and the animals were allowed to run on a 60 cm-long and 7 cm-wide track covered with white paper. A dark chamber was placed at the end of the track to entice rats. Upon completion of the test, the paper was scanned at 300 dpi. The measurement around the paw was defined as paw area (cm²), the distance between the first and fifth toes as paw width (cm), the distance of the same hind paw between two steps as stride length (cm), the horizontal distance between the left and right paw as the base (cm), the distance between the third toe and the heel as paw length (cm) and the paw angle as the angle through the hind legs (°). The measures of footsteps were quantified by ImageJ software (version 1.43u, National Institutes of Health, USA).

### Western Blotting

Protein expression levels of TNF-α, IL-1β, IL-6, COX-2, 5-lipoxygenase (5-LOX), matrix metalloproteinase-3 (MMP-3), and NFκB in joint tissue samples were analyzed by western blotting. The hind paws were quickly harvested and frozen at −80°C. The joint tissue homogenates were prepared in ice-cold lysis buffer containing 50mM Tris-HCl (pH, 8.0), 5mM EDTA, 0.26% sodium deoxycholate, 1% Triton X-100, 150mM NaCl, 50mM sodium fluoride, 10mM α-glycerophosphate, 0.1mM sodium orthovanadate, 50 μg/ml phenylmethylsulfonyl fluoride, and 10 μg/ml leupeptin. The homogenates were incubated on ice for 40 min. Sodium dodecyl sulfate-polyacrylamide gel electrophoresis (SDS-PAGE) sample buffer containing 2% β-mercaptoethanol was added to the supernatant. Twenty micrograms of protein were electrophoresed and then transferred into nitrocellulose membranes (Schleicher and Schuell Inc., Keene, NH, USA). Nitrocellulose blots were washed in phosphate-buffered saline (PBS) for 5 min and blocked with 1% bovine serum albumin in PBS for 1 h prior to administration of the primary antibodies (TNF-α, IL-1β, IL-6, COX-2, 5-LOX, MMP-3, and NFκB) (Abcam, Cambridge, UK)) that were diluted (1:1000) in the same buffer containing 0.05% Tween-20. The nitrocellulose membrane was incubated with the primary antibodies overnight at 4°C. The blots were washed and incubated with goat anti-mouse IgG antibody, HRP conjugate (Abcam, Cambridge, UK). Specific binding was detected using hydrogen peroxide and diaminobenzidine, as substrates. Protein loading was checked using an antibody against β-actin (A5316; Sigma Aldrich, St. Louis, MO, USA). Blots were performed at least three times to confirm the reproducibility of results. Bands were analyzed densitometrically using an image analysis system (Image J; National Institute of Health, Bethesda, USA).

### Statistical Analysis

**Figure 2:**
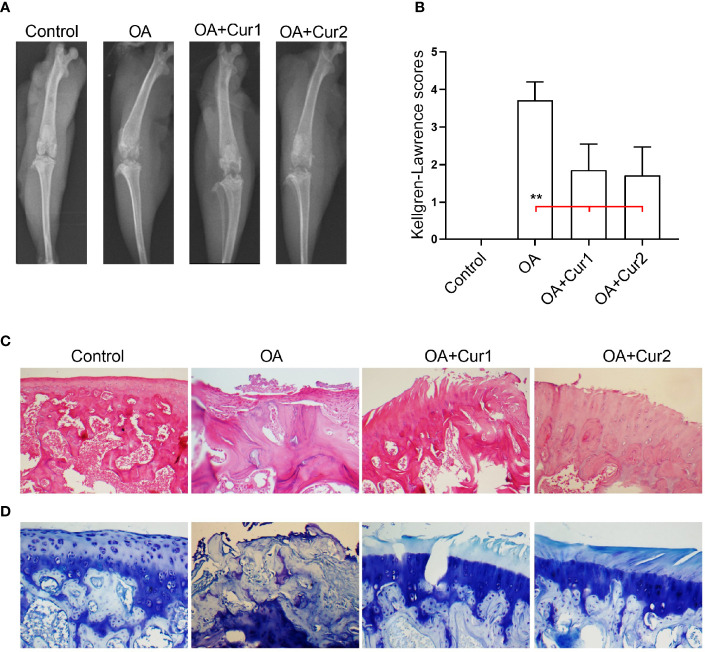
Radiological and histological assessment of the knee joints in rats with monosodium iodoacetate (MIA)-induced OA after Next Generation Ultrasol Curcumin (NGUC) supplementation. **(A)** Representative radiographic images obtained at the end of the experiment are shown. **(B)** Mean values of Kellgren-Lawrence scores are demonstrated with ± SD (n=7 for each group). **(C)** Representative histopathological images of the hematoxylin and eosin staining in the indicated group of rats. **(D)** Representative histopathological images of the toluidine blue staining in the indicated group of rats. Statistical differences of the Kellgren-Lawrence scores among the groups were determined by a Kruskal-Wallis followed by the Mann-Whitney U test; ^**^ *p* < 0.01. NGUC, next generation ultrasol curcumin; OA, osteoarthritis; OA+Cur 1, 100 mg/kg of NGUC; OA+Cur 2, 200 mg/kg of NGUC.

SPPS statistical package program (IBM SPSS Version 22.0) was used for statistical data analysis. One-way analysis of variance (ANOVA) test followed by a *post-hoc* Tukey test was used to determine the differences between the groups. A Kruskal-Wallis followed by the Mann-Whitney U test was used for data in **Figure 2**. Statistical significance was taken when *P*<0.05.

## Results

### Enhanced Bioavailability of NGUC Compared to 95% Turmeric Extract

**Figure 1:**
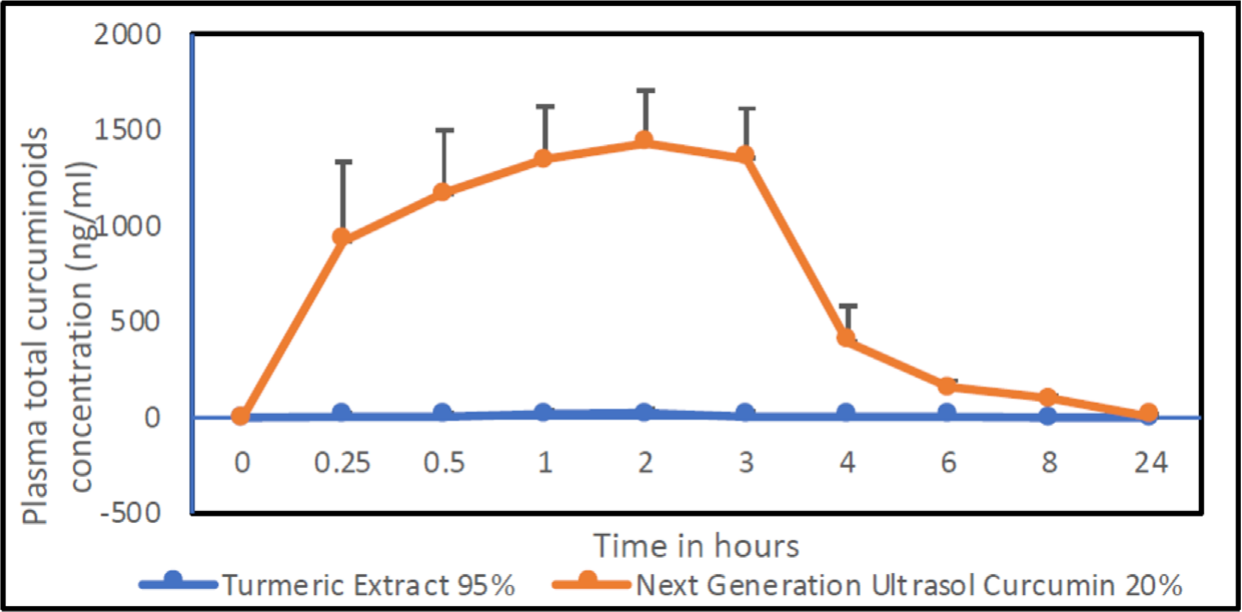
Plasma concentration of total curcuminoids vs. time profiles after oral administration of NGUC and 95% turmeric after dosing aqueous suspension of equivalent amount of total curcuminoids (dose 200 mg/kg body weight). The data are presented as means ± SD (n=8).

We measured plasma concentrations of total curcuminoids comprising of curcumin, demethoxycurcumin and bisdemethoxycurcumin after single-dose administration of 1,000 mg/kg body weight of NGUC (corresponding to 200 mg/kg body weight of curcuminoids) and 210 mg/kg body weight of 95% turmeric extract (corresponding to 200 mg/kg body weight of curcuminoids) to male Sprague Dawley rats. We observed for NGUC compared to 95% turmeric extract 54 folds higher Cmax, 64.7 folds higher AUC0-last, 2.9 folds higher Tmax, and 2.3 folds higher T1/2 (**Table 1**). The plasma concentration of total curcuminoids was significantly higher in rats administered with NGUC compared to 95% turmeric extract (**Figure 1**).

**Table 1:** Pharmacokinetic parameters after oral administration of NGUC and 95% turmeric extract as an aqueous suspension (dose 200 mg/kg body weight of total curcuminoids).

	Turmeric Extract 95%	NGUC 20%	Fold increase of NGUC/Turmeric extract 95%
C_max_ (ng/ml)	28.169 ± 28.604	1522.184 ± 257.839	54.04X
T_max_ (h)	0.656 ± 0.719	1.938 ± 0.863	2.95X
AUC_last_ (h*ng/ml)	95.349 ± 55.134	6170.474 ± 1071.967	64.71X
T_1/2_ (h)	2.190 ± 0.810	5.103 ± 0.725	2.33X

The data are presented as means ± SD (n=8). NGUC, next generation ultrasol curcumin; C_max_, maximum concentration; T_max_, time to reach maximum concentration; AUC_last_, the area under the concentration; T_1/2_, half-life time.

### The Effect of NGUC Supplementation on Serum Biochemical Parameters

We next measured serum biochemical parameters in the animals to assess the safety profile of NGUC supplementation and found that the induction of OA by the administration of MIA or subsequent treatment with NGUC did not change the overall biochemical components of the serum such as glucose, creatinine, BUN, TP, ALB, GLOB, ALT, AST, ALP, and TBIL (**Table 2**).

**Table 2:** Effects of two different doses of NGUC supplementation on biochemical parameters in the serum of rats with monosodium iodoacetate (MIA)-induced OA.

	Groups			
	Control	OA	OA+Cur1	OA+Cur2
Glucose, mg/dl	111.43 ± 14.79	113.57 ± 9.74	114.86 ± 7.06	110.57 ± 10.29
Creatinine, mg/dl	0.42 ± 0.16	0.42 ± 0.10	0.39 ± 0.14	0.41 ± 0.14
BUN, mg/dl	23.16 ± 1.9	21.64 ± 1.45	22.1 ± 0.76	21.89 ± 2.27
TP, g/dl	6.91 ± 0.65	7.29 ± 0.70	7.47 ± 0.57	7.67 ± 0.20
ALB, g/dl	3.71 ± 0.41	3.70 ± 0.29	3.97 ± 0.21	3.89 ± 0.35
GLOB, g/dl	3.41 ± 0.27	3.51 ± 0.23	3.53 ± 0.37	3.59 ± 0.24
ALT, U/L	74.14 ± 6.2	72.14 ± 4.88	70.29 ± 6.58	71.29 ± 7.16
AST, U/L	122.14 ± 8.76	120.29 ± 9.32	123.86 ± 10.79	122.43 ± 10.08
ALP, U/L	157 ± 21.92	153 ± 17.05	154 ± 33.05	158.29 ± 32.2
TBIL, mg/dl	0.20 ± 0.01	0.21 ± 0.01	0.24 ± 0.02	0.24 ± 0.01

The data are presented as means ± SD (n=7). Statistical differences among the groups were determined by one-way ANOVA followed by a post-hoc Tukey test (p > 0.05). NGUC, next generation ultrasol curcumin; OA, osteoarthritis; OA+Cur 1, 100 mg/kg of NGUC; OA+Cur 2, 200 mg/kg of NGUC. BUN, blood urea nitrogen; TP, total protein; ALB, albumin; GLOB, globulin; ALT, alanine aminotransferase; AST, aspartate aminotransferase; ALP, alkaline phosphatase; TBIL, total bilirubin.

### NGUC Supplementation Decreases the Severity of MIA-Induced OA in Rats

In order to test the effects of NGUC on the pathogenesis of OA, we evaluated the radiological features of the knee joint in rats with MIA-induced OA and subsequent supplementation with NGUC using Kellgren-Lawrence grading system (27). X-ray images of the knee joint revealed significant pathological changes in animals with MIA-induced OA compared to the control group of animals. Interestingly, NGUC supplementation significantly reduced the MIA-induced abnormalities in rats (**Figures 2A, B**). Histopathological analysis after H&E staining revealed extensive degeneration of joint architecture with a marked increase in inflammatory cell infiltration in MIA-induced OA, and these abnormal features were considerably reduced by NGUC supplementation (**Figure 2C**). Degradation of the articular cartilage induced by MIA injection was ameliorated in rats supplemented with NGUC as examined after toluidine blue staining (**Figure 2D**).

**Figure 3:**
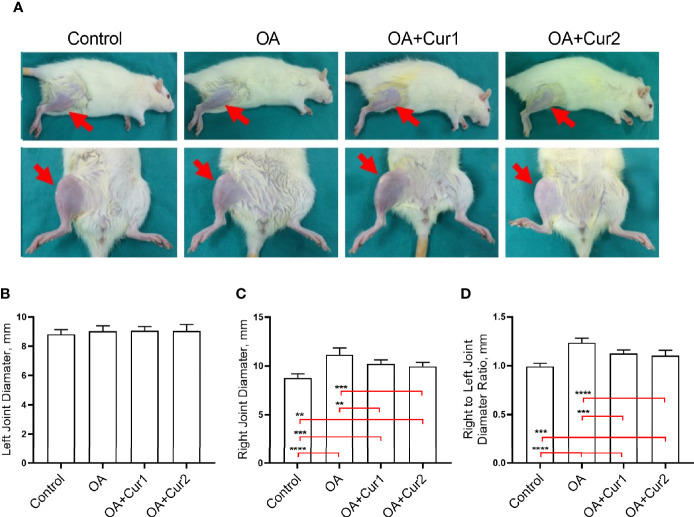
Assessment of the knee swelling in rats with monosodium iodoacetate (MIA)-induced OA after NGUC supplementation. **(A)** Images of the hind knee joints 4 weeks after Next Generation Ultrasol Curcumin (NGUC) supplementation in rats with MIA-induced OA. Graphs demonstrate **(B)** left knee joint diameter, **(C)** right knee joint diameter and **(D)** the ratio of right to left diameter values in rats with MIA-induced OA. The data are presented as means ± SD (n=7 for each group). Statistical differences among the groups were determined by one-way ANOVA followed by a *post-hoc* Tukey test; ^**^ *p* < 0.01, ^***^ *p* < 0.001, ^****^ *p* < 0.0001. NGUC, next generation ultrasol curcumin; OA, osteoarthritis; OA+Cur 1, 100 mg/kg of NGUC; OA+Cur 2, 200 mg/kg of NGUC.

We then compared MIA-induced knee swelling by measuring the left and right joint diameters. As illustrated in **Figure 3**, there was a significant increase of right joint diameters in rats with MIA-induced OA compared to the control group, while left joints remained unaffected. Four weeks of NGUC supplementation significantly reduced right joint swelling compared to the OA group (**Figures 3A–D**).

**Figure 4:**
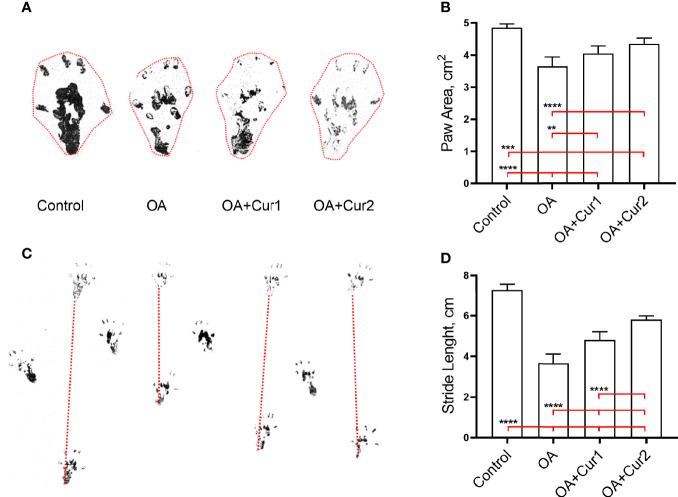
Effects of Next Generation Ultrasol Curcumin (NGUC) supplementation on paw area and stride length in rats with monosodium iodoacetate (MIA)-induced OA. **(A)** Representative measurement of paw area. **(B)** Graph shows paw area presented as cm^2^ in indicated group of rats. **(C)** Representative measurement of stride length. **(D)** Graph shows stride length presented as cm in indicated group of rats. The data are presented as means ± SD (n=7 for each group). Statistical differences among the groups were determined by one-way ANOVA followed by a *post-hoc* Tukey test; ^**^ *p* < 0.01, ^***^ *p* < 0.001, ^****^ *p* < 0.0001. NGUC, next generation ultrasol curcumin; OA, osteoarthritis; OA+Cur 1, 100 mg/kg of NGUC; OA+Cur 2, 200 mg/kg of NGUC.

Induction of OA in rats is associated with alterations in gait and we tested whether this could be improved by NGUC supplementation. We found that the injection of MIA caused a reduction in paw area and stride length, and NGUC supplementation partially reversed these features with NGUC at dose 200 mg/kg (40 mg/kg of curcuminoids) body weight performing better than 100 mg/kg (20 mg/kg of curcuminoids) body weight (**Figures 4A–D**). Collectively, our results suggest that NGUC supplementation ameliorates the disease severity of MIA-induced OA in rats.

### NGUC Regulates Inflammatory Mediators in Rats With MIA-Induced OA

As increased levels of COMP and CRP has been observed in various diseases, including OA (28–32), we next determined the levels of COMP and CRP after NGUC administration. We observed increased serum levels of COMP and CRP in rats with MIA-induced OA, which was significantly reduced by NGUC supplementation (**Table 3**). We also performed an ELISA for the detection of multiple inflammatory cytokines in the serum of rats. We found an increase in serum levels of pro-inflammatory cytokines TNF-α, IL-1β, IL-6, and a decrease in the level of anti-inflammatory cytokine IL-10 in MIA-induced OA compared to normal animals. Importantly, NGUC supplementation reduced levels of pro-inflammatory cytokines (IL-1β, IL-6, and TNF-α) and increased the level of IL-10 in the serum of rats compared to the OA group (**Table 3**).

**Table 3:** Effects of NGUC supplementation on serum inflammation markers in rats with monosodium iodoacetate (MIA)-induced OA.

	Groups			
	Control	OA	OA+Cur1	OA+Cur2
COMP, ng/ml	7.77 ± 0.88	27.68 ± 2.99^***^	20.11 ± 2.08^***^,###^^	14.44 ± 1.98^***,###,+++^
CRP, mg/L	1.25 ± 0.33	11.27 ± 1.55^***^	5.81 ± 0.70^***,###^	4.21 ± 0.84^***,###,+^
IL-1β, pg/ml	18.61 ± 1.67	54.79 ± 4.64^***^	41.03 ± 2.96^***,###^	31.96 ± 2.62^***,###,+++^
IL-6, pg/ml	10.58 ± 1.02	63.93 ± 5.38^***^	48.40 ± 3.91^***,###^	36.93 ± 2.90^***,###,+++^
IL-10, pg/ml	98.12 ± 6.33	34.06 ± 2.61^***^	47.58 ± 4.12^***,###^	57.09 ± 6.39^***,###,+^
TNF-α, pg/ml	21.29 ± 3.14	72.05 ± 8.61^***^	55.48 ± 7.93^***,##^	43.71 ± 6.90^***,###,+^

The data are presented as means ± SD (n=7). Statistical differences among the groups were determined by one-way ANOVA followed by a post-hoc Tukey test; ***p < 0.001 as compared to control group, ^##^p < 0.01, ^###^p < 0.001 as compared to OA group, and ^+^p < 0.05, ^+++^p < 0.001 as compared to OA+Cur1 group. NGUC, next generation ultrasol curcumin; OA, osteoarthritis; OA+Cur 1, 100 mg/kg of NGUC; OA+Cur 2, 200 mg/kg of NGUC; COMP, cartilage oligomeric matrix protein; CRP, C-reactive protein; IL, interleukin; TNF-α, tumor necrosis factor alpha.

**Figure 5:**
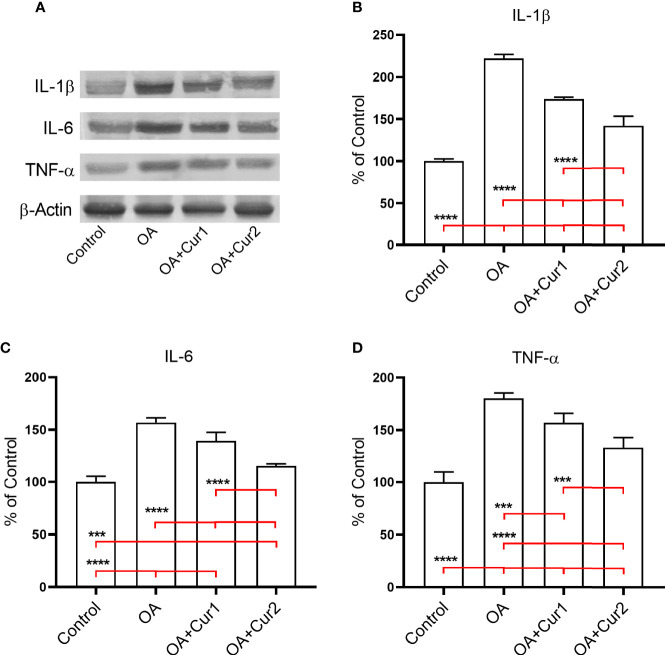
Effects of Next Generation Ultrasol Curcumin (NGUC) supplementation on protein expressions of pro-inflammatory mediators in rats with monosodium iodoacetate (MIA)-induced OA. **(A)** Representative protein expressions of IL-1β, IL-6 and TNF-α levels in synovial tissue of rats. β-actin was referenced to ensure equal protein loading. Graphs demonstrate densitometric analysis of the western blots for **(B)** IL-1β, **(C)** IL-6 and **(D)** TNF-α. Data represent percent of the control values. Blots were repeated at least three times. Statistical differences among the groups were determined by one-way ANOVA followed by a *post-hoc* Tukey test; ^***^ *p* < 0.001, ^****^ *p* < 0.0001. NGUC, next generation ultrasol curcumin; OA, osteoarthritis; OA+Cur 1, 100 mg/kg of NGUC; OA+Cur 2, 200 mg/kg of NGUC; IL, interleukin; TNF-α, tumor necrosis factor alpha.

We also tested protein levels of IL-1β, IL-6, and TNF-α in the synovial tissue of rats by western blotting. Increased levels of pro-inflammatory cytokines IL-1β, IL-6, and TNF-α were also observed in the synovial tissue of MIA-induced OA rats, which was significantly reversed by NGUC supplementation (**Figures 5A–D**). Notably, NGUC at 200 mg/kg (40 mg/kg of curcuminoids) had better biological effects compared to 100 mg/kg (20 mg/kg of curcuminoids) body weight dose (**Table 3** and **Figures 5A–D**). Collectively, these results suggest that NGUC has anti-inflammatory effects on MIA-induced OA in rats.

### NGUC Ameliorates Oxidative Stress in Rats With MIA-Induced OA

As oxidative stress plays a critical role in the pathogenesis of OA (2, 33), we hypothesized that NGUC may also affect oxidative stress signaling in OA induced by MIA-injection in rats. To test this possibility, we first measured serum MDA levels as a marker of oxidative stress. In agreement with the findings in OA patients (34), induction of OA by MIA-treatment resulted in an increase in the level of MDA compared to the control group of rats (**Table 4**). This phenotype was however partially reversed by NGUC administration (**Table 4**). We also evaluated the levels of antioxidant enzymes SOD, CAT, and GPX in the serum of rats, and observed reduced serum anti-oxidant levels in MIA induced OA. The reduction in the levels of antioxidant enzymes was partially but significantly corrected by NGUC with a better response in case of a higher dose of NGUC (**Table 4**). These data suggest a critical role for NGUC supplementation in the regulation of oxidative stress markers in OA pathogenesis.

**Table 4:** Effects of NGUC supplementation on serum levels of MDA and antioxidant enzymes in rats with monosodium iodoacetate (MIA)-induced OA.

	Groups			
	Control	OA	OA+Cur1	OA+Cur2
MDA, nmol/ml	0.63 ± 0.07	1.97 ± 0.08^***^	1.65 ± 0.09^***,###^	1.19 ± 0.18^***,###,+++^
SOD, U/ml	50.45 ± 4.07	21.86 ± 3.43^***^	31.35 ± 2.35^***,###^	38.03 ± 3.59^***,###,++^
CAT, U/ml	142.68 ± 6.58	60.64 ± 7.48^***^	71.76 ± 5.74^***,#^	89.77 ± 6.05^***,###,+++^
GPx, U/ml	117.94 ± 5.68	59.82 ± 3.32^***^	63.78 ± 6.69^***^	85.84 ± 4.98^***,###,+++^

The data are presented as means ± SD (n=7). Statistical differences among the groups were determined by one-way ANOVA followed by a post-hoc Tukey test; ***p < 0.001 as compared to control group, ^#^p < 0.05, ^###^p < 0.001 as compared to OA group, and ^++^p < 0.01, ^+++^p < 0.001 as compared to OA+Cur1 group. NGUC, next generation ultrasol curcumin; OA, osteoarthritis; OA+Cur 1, 100 mg/kg of NGUC; OA+Cur 2, 200 mg/kg of NGUC; MDA, malondialdehyde; SOD, superoxide dismutase; CAT, catalase; GPx, glutathione peroxidase.

### NGUC Supplementation Reversed MIA-Induced Protein Expressions of MMP-3, COX-2, 5-LOX, and NFκB in Rats

**Figure 6:**
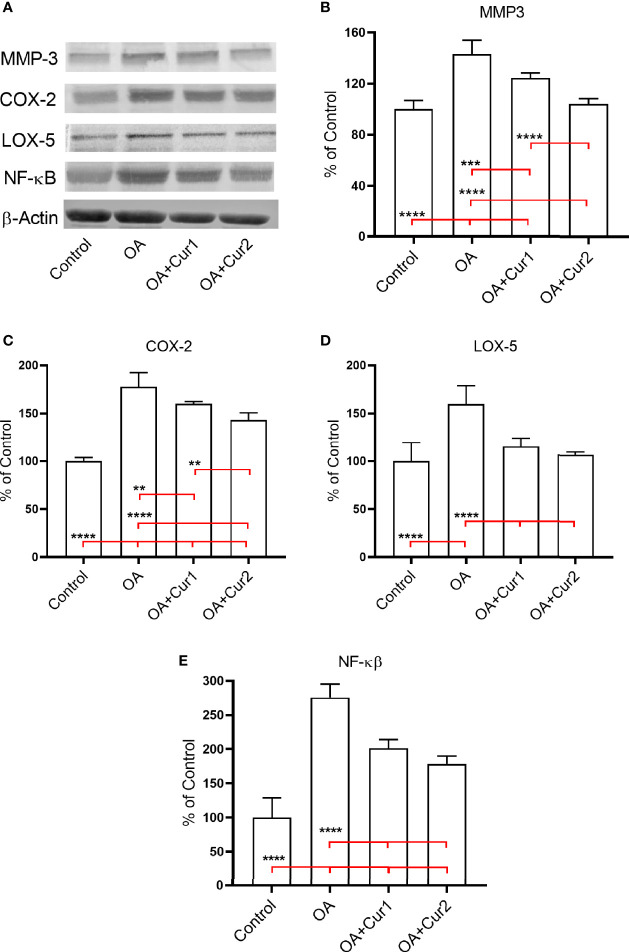
Effects of Next Generation Ultrasol Curcumin (NGUC) supplementation on protein expressions of MMP-3, COX-2, 5-LOX, and NFκB in rats with monosodium iodoacetate (MIA)-induced OA. **(A)** Representative protein expressions of MMP-3, COX-2, 5-LOX, and NFκB levels in synovial tissue of rats. β-actin was referenced to ensure equal protein loading. Graphs demonstrate densitometric analysis of the western blots for **(B)** MMP-3, **(C)** COX-2, **(D)** 5-LOX, and **(E)** NFκB. Data represent percent of the control values. Blots were repeated at least three times. Statistical differences among the groups were determined by one-way ANOVA followed by a *post-hoc* Tukey test; ^**^ *p* < 0.01, ^***^ *p* < 0.001, ^****^ *p* < 0.0001). NGUC, next generation ultrasol curcumin; OA, osteoarthritis; OA+Cur 1, 100 mg/kg of NGUC; OA+Cur 2, 200 mg/kg of NGUC; MMP-3, matrix metalloproteinase-3; COX-2, cyclooxygenase-2; 5-LOX, 5-lipoxygenase; NFκB, Nuclear Factor kappa B.

MMP-3 contributes to the destruction of cartilage by degrading various components of extracellular matrix and collagens, and high expression of MMP-3 has been identified in patients with OA (35). We measured the protein expression of MMP-3 in the synovial tissue of rats by western blotting and found an increased level of MMP-3 in rats with MIA-induced OA, and NGUC supplementation reduced this level with a better response in animals receiving higher dose (**Figures 6A, B**).

Similarly, we found increased expression of pro-inflammatory enzymes COX-2 and 5-LOX in synovial tissues of MIA-induced OA in rats compared to the control group. Four weeks NGUC administration after MIA-injection downregulated protein expressions of COX-2 and 5-LOX in synovial tissues of rats with MIA-induced OA (**Figures 6A, C, D**).

The transcription factor NFκB controls signaling events that mediate inflammatory responses and we found an increase in expression of NFκB in MIA-induced OA (**Figures 6A, E**). Similar to the results presented above, NGUC supplementation partially corrected NFκB expression with a better result at a higher dose (**Figures 6A, E**). These results collectively suggest that NGUC supplementation alleviates the severity of OA by down-regulating protein expressions of MMP-3, COX-2, 5-LOX, and NFκB in the synovial tissue of rats with MIA induced OA.

## Discussion

In the present study, we developed a new formulation of curcumin, called NGUC, with improved oral bioavailability as determined by pharmacokinetic studies conducted in rats. We further explored the role of NGUC supplementation in the pathogenesis of OA and showed that NGUC administration alleviates disease severity in rats with MIA-induced OA. The positive effects of curcumin on experimentally induced OA in rats were found to be associated with inflammatory mediators as well as oxidative signaling markers.

Curcumin is one of the most extensively studied natural products with a broad range of biological activities and therapeutic potential. However, curcumin has uncertain clinical efficacy due to its low bioavailability and high metabolism in the gastrointestinal tract. Curcumin is a hydrophobic molecule and insoluble in water (36) and has a short half-life at physiological pH due to high instability in alkaline pH (37). Even with high doses of curcumin supplement, very low plasma curcumin levels were detected for conventional curcumin (38), and hence the therapeutic potential of curcumin remains a concern. An increase in oral bioavailability is expected to directly influence plasma concentration as well as the therapeutic effects of curcumin. We used a combination of established excipients, natural phospholipids and monoglyceride to improve solubility and intestinal absorption, acidifiers to protect the microenvironment of curcumin in the alkaline pH of the small intestine, hypromellose and medium-chain triglyceride oil in our formulation to enhance bioavailability. Data from a pharmacokinetic study in rats indicated that after oral administration of 1000 mg/kg (200 mg equivalent of total curcuminoids) in the form of our new formulation, NGUC resulted in a peak serum concentration which was 64.7-fold higher as compared to natural 95% turmeric extract which indicated a significant increase in absorption of curcumin. As medium-chain triglycerides have anti-inflammatory activities, it is important to note that the total concentration of medium-chain triglycerides used in the preparation was only 1%, and at this low concentration they may not contribute significantly to the measured biological effects of NGUC.

We further conducted an efficacy study in a model of MIA-induced OA in rats which is a well-established animal model for OA to determine the protective effect of NGUC. While there are other animal models for the study of OA pathogenesis, the MIA-induced OA is suggested to be one of the best models to test the pharmacological effects and clinical efficacy of new pharmacological drugs (39). Injection of MIA into joints inhibits glyceraldehye-3-phosphate dehydrogenase activity in chondrocytes, leading to disruption of glycolysis and cell death. Further, MIA-induced OA in rats resembles human OA with similar histological features and pain-related behaviors (40–42). OA affects all facets of joint integrity, with thinning of the articular cartilage accompanied by subchondral bone thickening, along with the formation of bony osteophytic projections (43). We find that NGUC alleviates the severity of OA by restoring the architecture of the joint. The development of knee OA promotes pain and people with OA have specific gait patterns for the adaptation to pain (44–46). OA patients have been suggested to have reduced stride length and walking speed (47–49). Therefore, the development of better therapeutics in the treatment of OA would increase the quality of life of patients. Similar to the gait alterations in patients, chemically-induced OA in animals also causes gait abnormalities (50). Strikingly, our findings revealed an improvement in paw area and stride length in rats with MIA-induced OA after NGUC supplementation, suggesting an additional positive effect of NGUC on gait abnormalities caused by OA.

OA is associated with dysregulated immune mediators (51) with increased levels of pro-inflammatory mediators that play a critical role in the pathophysiology of the disease (52). The increased serum COMP and CRP levels in response to the underlying inflammatory condition is used as a biomarker for arthritis, including OA (22–26). We found increased levels of COMP and CRP in MIA-induced OA, which was reversed by NGUC. Matrix metalloproteinases such as MMP-3 play a significant role in the destruction of cartilage by degrading various components of extracellular matrix and collagens, and high expression of MMP-3 has been observed in patients with OA (35). NGUC treatment also reduced serum MMP3 levels in MIA-induced rats.

Pro-inflammatory enzymes such as COX-2 and 5-LOX play a vital role in the pathogenesis of OA and increased levels of these enzymes in synovial tissues of OA and RA patients have been observed (32). 5-LOX inhibitors have been shown to reduce TNF-α-induced inflammation in human synovial fibroblast (53). Similarly, NSAIDs used for the treatment of OA target COX-2 (54). However, although the use of selective inhibitors targets inflammatory mediators, adverse events of those inhibitors may also be possible (55). Thus, there is a need for the development of more targeted drugs with no or less adverse effects in the treatment of OA. In addition to conventional treatment strategies, consumption of bioactive compounds including antioxidants, vitamins, minerals, and flavonoids offers a treatment option and may exert beneficial effects against OA by selectively targeting inflammatory mediators. We demonstrate that NGUC with no adverse effects mediates the inflammatory pathways associated with COX-2 and 5-LOX and reduces the severity of OA.

Oxidative stress plays an important role in the pathogenesis of OA (2, 33). Oxidative stress is an outcome of an imbalance between the formation of reactive oxygen species and antioxidant defense mechanisms accomplished *via* various molecules and enzymes such as SOD, CAT, and GPX (2). Our results showed decreased serum levels of antioxidant enzymes in MIA-induced OA in rats as seen in OA patients (56). However, NGUC supplementation increased the levels of SOD, CAT, and GPX, suggesting a further role for NGUC in the regulation of antioxidant enzymes in the progression of OA.

Since various pathways have been linked to the pathophysiology of OA, a key question arising from our results is how NGUC supplementation attenuates the severity of OA in rats. Several studies have reported that the treatment of human articular chondrocytes with curcumin inhibited IL-1β and TNF-α-induced NFκB activation, resulting in downregulation of COX-2 (57). Similarly, curcumin promoted inhibition of IL-1β-induced IL-6 and MMP-3 production by human chondrocytes (58) and extracellular matrix degradation (59). Moreover, curcumin supplementation decreased mRNA expressions of pro-inflammatory mediators, including IL-1β, TNF-α and MMP-3 in a post-traumatic OA mouse model (60). Using a microarray analysis for the differentially expressed genes in synovial tissue of OA, Zeng et al. observed inhibition of MMP-3 expression in the OA group treated with curcumin compared with the untreated group (61). It has also been shown that curcumin supplementation reduces disease progression in the destabilization of the medial meniscus surgical instability model of OA by suppressing both the expressions of pre-inflammatory mediators and activation of NLRP3 inflammasome (62). Interestingly, the suppression of NLRP3 inflammasome by curcumin supplementation has also been observed in lupus-prone mice (63). Moreover, curcumin has also protective effects against OA in mice by inducing autophagy *via* AKT/mTOR pathway (64) and blocking TLR4/MyD88/NFκB signaling pathway (65). In consistent with these findings, we observed that NGUC treatment reduced MMP-3 expression reflecting the inhibition of cartilage destruction in rats. Moreover, we found a significant correction of OA pathogenesis by NGUC through modulation of pro-inflammatory cytokines. Furthermore, NGUC reduced MIA-induced NFκB and its target COX-2. Thus, we conclude that NGUC modulates multiple pathways that include mediators of the pathogenesis of OA such as NFκB, COX-2, MMP-3, COMP, CRP and ameliorates overall pathology.

The protective effects of curcumin on the progression of OA have also been reported in humans. A curcumin-phosphatidylcholine complex reduced the levels of inflammation markers such as IL-1β and IL-6 in the serum of OA patients (66). Moreover, nanomicelle curcumin improved the symptoms of OA patients (67). Shep et al. compared the effects of curcumin on knee OA against diclofenac, a drug used to relieve OA pain and stiffness (68), and reported a similar improvement in the severity of pain with less adverse effects by curcumin (69). The serum levels of MDA and SOD in OA patients have also been increased by curcumin supplementation (56). More recently, a study revealed that curcumin supplementation alleviates the severity of knee OA in humans by regulating different immune cell subsets (70). The authors showed that curcumin administration reduced CRP level and caused a reduction in the percentage of CD4^+^, CD8^+^ and Th17^+^ T cells, and a concomitant increase in the percentage of regulatory T cells in OA patients (70). Therefore, the findings of our study as well as others performed in animal models suggest that the positive effects of curcumin supplementation on the pathogenesis of OA may possibly have a corresponding effect in humans. Hence, we believe that therapeutic effect of NGUC could further accentuate in OA due to its increased oral bioavailability and higher plasma levels.

In conclusion, our findings indicate that a novel and highly bioavailable formulation of curcumin, NGUC decreases the severity of MIA-induced OA in rats, and the protective effect of NGUC is mediated *via* modulation of inflammation mediators and oxidative stress markers. In addition to conventional therapies, we believe that NGUC may offer a safe option for the treatment of OA in humans, which should be validated through carefully designed studies. The human studies may include the comparison of NGUC with NSAIDs and/or corticosteroids that are generally used in the management of OA symptoms, which is a limitation of our study. However, it should be noted that although the MIA model of OA has been widely used for the study of OA pathogenesis it may not perfectly reflect human OA, and has several limitations including the rapid development of OA in the animal model compared to the development of OA in a long time period in humans.

## Data Availability Statement

The original contributions presented in the study are included in the article/supplementary material. Further inquiries can be directed to the corresponding author.

## Ethics Statement

The animal study was reviewed and approved by Institutional Animal Ethics Committee (IAEC Protocol No.: ABD/IAEC/PR/89/17-20) and Animal Ethics Committee of Firat University (2019/88-135).

## Author Contributions

MY designed the study, analyzed data, and wrote the paper. CO performed experiments and analyzed data. BE performed experiments. MT performed experiments and analyzed data. AD performed the radiological assessment. IO performed the histological assessment. NS designed the study and analyzed data. PB prepared NGUC formulation. AM provided and edited manuscript. MP provided and edited manuscript. KS designed the study, analyzed data, and wrote the paper. All authors contributed to the article and approved the submitted version.

## Funding

OmniActive Health Technologies and TUBITAK (KS). The funders had no role in the project design, data collection, data analyses and interpretation.

## Conflict of Interest

PB, AM, and MP are employees of OmniActive Health Technologies.

The remaining authors declare that the research was conducted in the absence of any commercial or financial relationships that could be construed as a potential conflict of interest.
